# Agri-Food Wastes as Natural Source of Bioactive Antioxidants—Third Edition

**DOI:** 10.3390/antiox14020198

**Published:** 2025-02-10

**Authors:** Silvana Hrelia, Maria Cristina Barbalace, Cristina Angeloni

**Affiliations:** Department for Life Quality Studies, Alma Mater Studiorum, University of Bologna, 47921 Rimini, Italy; silvana.hrelia@unibo.it (S.H.); maria.barbalace2@unibo.it (M.C.B.)

The current food systems are now unsustainable due to population growth, globalization, and climate change, contributing to environmental degradation and social inequalities [1]. The significant waste and losses generated at every stage of the food supply chain are one of the main factors contributing to food unsustainability, causing resource depletion, global greenhouse gas emissions, and environmental degradation. Therefore, reducing food waste is one of the most important strategies for sustainability [2]. The possibility of converting by-products and waste materials into high-value molecules could create a pathway to reduce environmental impact while promoting innovation and economic growth. In particular, agri-food waste includes a wide range of discarded parts from crops, fruits, and vegetables generated at different stages of the agricultural food supply chain, such as farming, harvesting, storage, and processing. These wastes include, but are not limited to, fruit skins, vegetable peels, seeds, stems, and other inedible or underutilized parts with little or no commercial value, and are commonly discarded despite their potential applications in other fields. The chemical composition of these food waste materials makes them a natural reservoir of bioactive compounds with potential health benefits for humans, because they are a source of phytochemicals that can be extracted and repurposed in nutraceutical, cosmeceutical, and pharmaceutical applications [3]. Recent studies have shown that fruit and vegetable by-products, which are traditionally discarded, present similar or even higher amounts of phytochemical compounds than edible tissue [4,5]. Particularly, polyphenols, classified as flavonoids, tannins, phenolic acids, stilbenes, and lignans, are widely present in waste products originating from fruits and vegetables. They are perhaps the most extensively studied class of bioactive compounds due to their antioxidant activity and ability to modulate inflammation as well as various signal transduction pathways [6,7]. The current literature suggests that the long-term consumption of diets rich in polyphenols protects against certain cancers, cardiovascular diseases, type 2 diabetes, and neurodegenerative diseases [8,9,10,11].

However, due to the limited water solubility and bioavailability of many phytochemicals, their pharmacological, nutraceutical, and cosmeceutical applications have been limited. Researchers have made progress in the development of nano- and micro-carriers that can facilitate effective delivery [12]. Optimizing the bioavailability of these molecules also depends on their efficient recovery. Thus, extraction procedures, especially from agri-food waste, play a crucial role in raising the total potency of these bioactive components. The increased consumer request for minimally processed, clean-label products enhances the necessity to apply more sustainable and specific extraction techniques [13].

The third edition of this Special Issue is primarily aimed at reviewing recent research, introducing new studies, and connecting these findings with today’s global health priorities, focusing on remedies and agents that enhance health and lower the risk of various diseases. Here, we have considered papers on the eco-green extraction and characterization of agri-food wastes to produce antioxidant bioactive compounds, as well as papers on in vitro and in vivo studies of the effects of these compounds/extracts, emphasizing the capacity of agri-food wastes to modulate the intricate signaling networks underpinning the development and progression of chronic and degenerative diseases.

This Special Issue includes two reviews and thirteen original articles that delve into recent research aimed at enhancing the functional potential of bioactive compounds, like phytochemicals, present in agri-food waste. These studies offer valuable insights into the effectiveness of food-waste-derived molecules as natural alternatives for managing health issues often associated with oxidative stress.

The description of new eco-friendly extraction techniques to obtain bioactive compounds from maize by-products (stalks, leaves, ears, and husks) is reported in the comprehensive review of Ramirez-Esparza et al. (Contribution 1). Traditional extraction methods that rely on the use of solvents are now increasingly being replaced by green methods that use ultrasound, microwave, and supercritical fluid extraction. Also, biotechnological approaches such as enzyme-assisted extraction and fermentation-assisted extraction are gaining attention. Both maize and its by-products (cob, maize hairs, and stover) contain bioactive phenolic compounds, which have been demonstrated to possess numerous health-protective properties. The review describes different techniques for separating and identifying the bioactive compounds, underlying the importance of the integral utilization of corn residues to obtain them, thus promoting agricultural sustainability and the development of products of added value in the food and pharmaceutical sectors.

Low-frequency ultrasound-assisted extraction was applied to apple pomace (the residue from apple pressing, consisting of peel, pulp, seeds, and stems) to simultaneously extract key antioxidant compounds like dihydrochalcones, quercetin glycosides, and triterpenic acids (Contribution 2). The extraction conditions were optimized using response surface methodology, and this method was also applied to analyze the components in whole apple pomace, apple peel, and apple flesh. Two linear multivariate regression models enabled the prediction of antioxidant activity in apple by-products based on their bioactive composition. The findings of this study highlight the potential of industrial cider apple pomace as a source of valuable bioactive compounds and demonstrate the practicality of ultrasound-assisted extraction as a simple and efficient recovery method.

Valorizing food by-products, such as spent brewer’s yeast and fruit pomaces, is a key strategy for promoting sustainable food production. The study of Dumitrascu et al. (Contribution 3) utilized the Maillard conjugates from spent yeast protein hydrolysate (SYH) combined with dextran (D) or maltodextrin (MD) through ultrasound treatment to develop encapsulation systems for anthocyanins from aronia pomace. The ultrasound-assisted Maillard reaction enhanced antioxidant activity compared to traditional heating and SYH alone. A freeze-drying method was used to evaluate the ability of the conjugates to serve as wall materials for encapsulating bioactive molecules, highlighting the influence of the carrier material on encapsulation performance. The inclusion of the hydrolyzed yeast cell wall positively affected the encapsulation efficiency of anthocyanins when combined with the SYH:MD conjugates. Conversely, the SYH:D conjugate showed better stability of anthocyanins during storage and higher bioavailability during gastrointestinal digestion. This study demonstrated the promising potential of combining hydrolysis with ultrasound-assisted Maillard reactions for the valorization of spent brewer’s yeast as a delivery material for encapsulating bioactive compounds.

The recovery of bioactive molecules from the primary by-product of citrus fruit processing, known as raw pomace or pastazzo, is of particular importance from a circular economy perspective due to the millions of tons of raw pomace produced annually. The research of Ingegneri et al. (Contribution 4) focused on the preparation of food-grade extracts from orange (OE) and lemon (LE) pomace through ultrasound-assisted maceration. After an initial phytochemical and biological screening using in vitro assays, the primary and secondary metabolites were identified using 1H-NMR and liquid chromatography coupled with diode array detection and electrospray ionization mass spectrometry (LC-DAD-ESI-MS). The plant-based complexes were tested for their antioxidant and anti-inflammatory properties by in vitro cell-free and cell-based assays to evaluate their potential usefulness for nutraceutical purposes in the context of inflammatory bowel disease (IBD). In particular, the intestinal bioaccessibility, antioxidant, and anti-inflammatory properties of the extracts were evaluated through in vitro-simulated gastrointestinal digestion, followed by testing on lipopolysaccharide (LPS)-stimulated human colorectal adenocarcinoma cells (Caco-2). The digests from OE and LE showed notable effects in protecting against LPS-induced intestinal barrier disruption, oxidative stress, and inflammatory responses, suggesting that both OE and LE are promising candidates for further preclinical studies in IBD models.

The study of Patra et al. (Contribution 5) is focused on the application of a quick, cost-effective, and eco-friendly method for producing gold nanoparticles (AuNPs) using papaya peel extract (VPPE) as a biowaste material. Papaya peel is a valuable source of phenolic compounds like apigenin, bemyricetin, kaempferol, quercetin, luteolin, and morin. Phytochemical analysis of the VPPE indicated the presence of tannins, saponins, steroids, proteins, and carbohydrates. The synthesized VPPE-AuNPs were characterized by different techniques, such as UV–Vis spectroscopy, X-ray diffraction, scanning electron microscopy–energy-dispersive X-ray, thermogravimetric analysis, and Fourier transform infrared spectroscopy. The study further examines the antioxidant, antidiabetic, tyrosinase inhibition, anti-inflammatory, antibacterial, and photocatalytic degradation activities of VPPE-AuNPs. The VPPE-AuNPs exhibited significant antioxidant activity, moderate tyrosinase inhibition, and strong α-glucosidase and α-amylase inhibition. Additionally, they were highly effective in degrading harmful dyes such as methyl orange and methylene blue. Overall, VPPE-AuNPs demonstrated multiple bioactive potentials that could be useful in food, cosmetic, and biomedical applications.

*Pyracantha*, commonly known as Firethorn, is an evergreen shrub of the Rosaceae family, native to regions characterized by a Mediterranean climate, such as Salento, a region in Southern Apulia in Italy. While this plant is typically grown as an ornamental one, previous studies demonstrated the diuretic, cardiac, and tonic properties of the fruits [14]. For the first time, the antioxidant activity, as well as the phenolic and anthocyanin composition of the extracts (obtained by two different extraction methods) from three varieties of *Pyracantha coccinea* and one variety of *Pyracantha angustifolia*, were examined and compared (Contribution 6). The data obtained represent a strong stimulus for the valorization of local plant resources, such as *Pyracantha* species, typical spontaneous flora of Southern Italy.

In line with current EU and global regulations aimed at reducing waste, improving its management, and especially focusing on its valorization, every secondary by-product from primary production (the waste) should be enhanced and put to productive use. The olive oil and wine industries generate tons of waste per year, so the development of new technologies that allow the recovery of waste and, in particular, of the bioactive compounds contained within would support the production of high-value products, contributing to the circular economy strategy. Most of the phenolic compounds found in olives do not transfer to the oil; instead, they remain in the olive pomace and olive vegetation water (OVW), which are the solid and liquid by-products, respectively, of the mechanical extraction process. The interesting paper of Mercatante et al. (Contribution 7) demonstrated that a phenolic extract produced from fresh OVW as spray-dried powder and added to minced beef meat can counteract the formation of cholesterol oxidation products and heterocyclic aromatic amines, which accumulate following high-temperature cooking methods (such as grilling and barbecuing) in red meat steaks and patties. Moreover, the mutagenicity and genotoxicity of stored cooked beef patties, both with and without phenols extracted from OVW, were evaluated using Mononuclear Cells from Peripheral Blood, demonstrating that the OVW phenols were able to counteract the formation of genotoxic compounds in stored cooked beef patties. Also, olive cake (OC), a major by-product of olive oil extraction, is particularly notable for its rich content of bioactive compounds and its potential for value-added recycling. The analysis of six OC samples collected from different mills in the Trás-os-Montes and Alto Douro regions (Portugal) at various processing stages demonstrated significant differences in the phenolic composition, particularly in ortho-diphenol and flavonoid concentrations across the samples, and these differences were reflected in the antioxidant activity (Contribution 8). Using HPLC-PDA-MS, 22 compounds were identified, with luteolin and verbascoside being particularly abundant. This comprehensive characterization underscores the potential of olive by-products for valorization, contributing to sustainability and supporting a circular economy within the olive oil industry. The study of Iervolino et al. (Contribution 9) aimed to characterize seed extracts of *V. vinifera* L. cv. Falanghina to analyze their phytochemical content, including total phenolic compounds, free radical scavenging capacities, and antioxidant activity, in HepG2 cells, with a particular focus on mitochondrial function. They demonstrated the ability of seed extracts to support mitochondrial biogenesis and mitophagy, improve mitochondrial efficiency, and reduce ROS production. These findings emphasize the potential of Vitis by-products to enhance the functional properties of foods.

Agri-food wastes, such as orange and lemon peels, rich in polyphenolic antioxidants, were utilized to develop hypoallergenic matrices formed by the interaction with hen egg proteins (specifically, ovalbumin and lysozyme) (Contribution 10). The protein–polyphenol interactions were characterized at the molecular level using IR monitoring, and the binding mechanisms were further simulated through docking analysis with lysozyme. The resulting edible matrices, which are inherently safe, show reduced IgE binding compared to the pure proteins in indirect immunological assays (ELISA) using serum samples from patients allergic to ovalbumin and lysozyme. This reduction in allergenicity may be attributed to the interactions with polyphenols, which modify the structure and functionality of the native proteins.

The disposal of animal by-products from milk poses a significant challenge for dairy companies due to their considerable environmental impact. Extracting bioactive components to create value-added foods from milk permeate (MP) (a by-product generated during the ultrafiltration process used to concentrate milk before fermentation for yogurt production) presents a promising circular strategy (Contribution 11). The peptide fractions isolated and identified through LC-MS/MS analysis demonstrated radical scavenging activity in vitro, and the ability to protect Caco-2 cells from oxidative stress by activating the Keap1/Nrf2 antioxidant signaling pathway. Moreover, the in vivo effects of the peptide fractions were also tested using a zebrafish model, where they protected the organism from the harmful effects of acute cold stress, highlighting the potential of MP antioxidant peptides for the development of functional foods.

Shrimp and crab shells are important sources of astaxanthin, together with *Haematococcus pluvialis*, with an annual biomass yield of hundreds of tons. The review of Dang et al. (Contribution 12) highlights the various forms of astaxanthin currently used as antioxidants in foods, including both its naturally occurring forms and artificially added ones, utilizing technologies such as emulsions, microcapsules, films, nano-liposomes, and nanoparticles, focused on increasing astaxanthin stability, dispersion, and bioavailability in complex food systems. The review summarizes the progress in applying astaxanthin to various food products, such as whole grains, seafood, and poultry. It is known that astaxanthin is water-insoluble and sensitive to light, heat, oxygen, and humidity. Therefore, the authors make interesting suggestions for developing astaxanthin-loaded systems with high encapsulation efficiency, good stability, and cost-effectiveness.

In the study of Liu et al. (Contribution 13), beet waste residues were fermented using *Leuconostoc pseudomesenteroides* to produce lactic acid bacteria exopolysaccharide (EPS), improving the utilization rate of this important agricultural waste and reducing the production cost. EPS was isolated, purified, and then tested for homogeneity, molecular weight, and monosaccharide composition. It demonstrated good thermal stability, specific viscosity, and oxidation resistance. Moreover, the protective effect of EPS toward mouse insulinoma 6 (MIN-6) cells treated with tert-butyl hydroperoxide was tested, evidencing a marked reduction in intracellular ROS levels and protection against oxidative stress. The results suggest the importance of using beet waste residues to obtain high-value-added products, with possible applications in the food and pharmaceutical industries.

The seeds and rinds of passion fruit, considered agricultural byproducts of juice processing, were repurposed to explore their biological activities for sustainable applications (Contribution 14). The extracts were characterized (Piceatannol and Scirpusin B were demonstrated to be the most representative molecules) and tested for neuroprotective activity in amyloid-β_25–35_-treated or H_2_O_2-_treated SH-SY5Y cells. The extracts evidenced neuroprotection against hydrogen peroxide-induced or Aβ_25–35_ peptide-induced cell death in SH-SY5Y cell models. To confirm the in vitro data, in vivo analyses on ICR mice were performed in order to demonstrate the extracts’ ability to improve learning and memory functions following scopolamine injection, in comparison to pure piceatannol and scirpusin. The recycled seeds and rinds of passion fruits from juice processing factories could represent a sustainable resource for developing functional foods aimed at addressing unmet medical needs, particularly in delaying the onset of cognitive dysfunction and neurodegenerative diseases.

Tomato skin and pomegranate peel are usually discarded. The paper from Silla et al. (Contribution 15) proposed cosmeceutical applications of tomato skin (HP) and pomegranate peel (PPE) extracts on oral mucosa to maintain oral health, evaluating their possible use in mouthwashes. Oral hygiene products represent a promising yet underexplored area for investigating the potential benefits of compounds derived from agri-food waste. Maintaining proper oral hygiene is essential for preventing and treating oral diseases; this emphasizes the need for effective oral care products like mouthwashes formulated with biologically active compounds with antioxidant, anti-inflammatory, and antibacterial properties to reduce the risk of dental decay and prevent the onset and progression of oral diseases. The biological activities of the extracts and the mouthwash containing them were evaluated in Human Primary Gingival Epithelial Cells treated with LPS, evidencing antioxidant and anti-inflammatory activities. Moreover, the antibacterial activity of the extracts and the mouthwash was evaluated against *Streptococcus mutans* and *Streptococcus sanguinis*, and both showed strong efficacy against these oral streptococcus species. The results highlight the potential of HP and PPE in reducing oxidative stress, inflammation, and bacterial growth in the oral mucosa, emphasizing food waste upcycling as a valuable resource for improving oral health.

The published scientific papers highlight the significance of utilizing the antioxidative properties of various vegetal and animal food waste. By exploring a diverse range of bioactive substances present in agri-food and animal waste, the authors emphasize the potential of upcycling to promote a circular economy and drive sustainable innovation in the food, pharmaceutical, and cosmeceutical industries. By transforming agri-food by-products into high-value ingredients, applying new ecocompatible techniques, the studies contribute to the development of environmentally mindful solutions for human health, tackling key challenges at the crossroads of sustainability and human well-being.
